# Biopreservation in Agriculture and Food Systems: A Summary of Ethical Issues

**DOI:** 10.1017/jme.2024.149

**Published:** 2024-12-16

**Authors:** Paul B. Thompson, John Bischof, Matthew J. Powell-Palm, Kieran Smith, Terrence R. Tiersch

**Affiliations:** 1. MICHIGAN STATE UNIVERSITY, EAST LANSING, MICHIGAN, USA.; 2. UNIVERSITY OF MINNESOTA, MINNEAPOLIS, MINNESOTA, USA.; 3. TEXAS A&M UNIVERSITY, COLLEGE STATION, TEXAS, USA.; 4. LOUISIANA STATE UNIVERSITY, BATON ROUGE, LOUISIANA, USA. AUTHORS AFTER THE FIRST ARE LISTED IN ALPHABETICAL ORDER.

**Keywords:** Cryopreservation, Agricultural Ethics, Emerging Technology, Assisted Reproduction In Food Animals, Isochoric Freezing, Agricultural Biorepositories

## Abstract

Biomedical research on advanced cryopreservation has spillover effects on innovation in the food and agricultural sector. Advanced biopreservation technology has three key domains of impact in the food system: (1) improving efficiencies in storage and utilization of gametes and organoids for plant and animal breeding; (2) isochoric methods for preservation of fresh food products; and (3) in biorepositories for storage of genetic resources for agriculturally significant plants and livestock species.

## Introduction

After a context-setting introduction, this paper identifies three applications of biopreservation in the food system, followed by a discussion of an ethical rationale for innovation in agriculture and food production that is applicable to each. Counters to this rationale include concerns about health risks, environmental impact, and ethically significant transitions in the socio-economic structure of farming, food processing, and the distribution of agricultural products. Applications in aquaculture provide an exemplary discussion of how these substantive ethical issues might arise in connection with biopreservation. However, recent controversy over gene technology suggests that substantive ethical concerns in agriculture may be less important than procedural questions about the manner in which ethical review is integrated into the research process, as well as key institutions’ responsiveness to substantive matters.

We will argue that biopreservation does not raise novel ethical issues in agriculture and food systems. The ethical rationale for biopreservation shares a structure with arguments for mechanization, chemical inputs, and biotechnology. In each case, advocates of technical change promise benefits in the form of more efficient methods that expand the food supply while reducing the environmental impact of farming. Yet, these technologies have also raised concerns seldom addressed by advocates of technological innovation in food systems. At the most general level, the history of unaddressed concerns gives rise to skepticism about the future benefits argument. Although biopreservation does not raise novel issues, innovators included questions about population growth and carrying capacity. Connections between biomedical and food or agricultural innovations would therefore be appropriate subjects for bioethical inquiry. Lee argues that since Potter’s time, bioethicists have narrowed the scope of their field in an unwarranted fashion, limiting capacity for engagement with ethicists working on environmental issues and public health.^2^ Calls for bioethics teaching and scholarship in agricultural universities were sounded not long after publication of Potter’s *Bioethics: A Bridge to the Future* in 1971. Yet while medical bioethics has blossomed into a multidisciplinary area of scholarship with programs in virtually every U.S. medical school, no similar development has occurred in agricultural universities.^3^ As we will argue at the conclusion of this paper, the weak institutional capacity for agricultural bioethics may be should not be sanguine. Continuing neglect and incapacity for bioethical studies in agriculture and food systems creates the potential for unexpected ethical and political controversy, so much so that this incapacity itself may be the most significant ethical hurdle for food or agricultural applications of biopreservation. Any technology following the tradition of ignoring ethical concerns risks inheriting pent-up resistance to technical change in food systems.

## I. The Context for Food and Agricultural Ethics

From a technical perspective, the research streams for biopreservation in medicine and in agricultural and environmental applications are not distinct. Research to develop tools in one area will aid further development in the other two. A thorough bioethics of advanced cryopreservation technology should address the full range of topics.^1^ As Lisa Lee argues, Van Renssellaer Potter’s original vision for bioethics the most serious ethical obstacle to innovative work in biopreservation.

Technological innovations in agriculture and medicine have long been deeply interconnected. Farm animal veterinarians performed techniques for artificial insemination long before they were attempted on humans. Costs for drug and vaccine development for food animals were substantially less than they might have been because of parallel developments in human medicine. Less favorably, the eugenics movement of the late 19th and early 20th century derived support from the experience of improving the performance of breeds used in animal agriculture.^4^ There is thus a rationale to ask whether research in either domain — medicine or agriculture — might have spillover effects that raise ethical questions in the other domain. In addition, agriculture and medicine are similar in that both non-profit research institutes (including universities) and for-profit firms perform scientific studies intended to result in novel tools and techniques for pursuing their respective ends. In both cases, tension between the profit motive and imperatives for safety and public benefit provides one motivation for training and inquiry in ethics. In agriculture, concern that industry had too much influence on public sector research was sounded following Rachel Carson’s disclosure of unwanted and understudied environmental and health effects of agricultural chemicals.^5^ By the 1980s, leading agricultural scientists and college administrators were calling for the integration of ethicists into the faculty of agricultural universities, the development of ethics courses for undergraduates and the integration of ethical analysis into the research process. While several colleges of agriculture initially took steps in that direction, conventional methods in applied economics and environmental sociology dominated research on ethically controversial topics. The impetus for building capacity for bioethics in agricultural research was on the wane by the end of the century.^6^

Weed scientist Robert Zimdahl has undertaken periodic canvasses of ethics programming at US agricultural universities. His findings demonstrate that teaching and research in bioethics (including agriculture and food law) were never developed in more than a fraction of agricultural colleges, and that the number of courses and faculty lines has decreased since a high point in about 1990.^7^ Explanations for this phenomenon vary. The prominent agricultural economist Glenn L. Johnson attributed it to the influence of philosophical positivism among agricultural scientists.^8^ Nutritionist Marion Nestle says industry interests have captured the agricultural sciences, undermining any attempt to even question the for-profit research agenda.^9^ Zimdahl himself argues for overweening moral confidence among agricultural researchers who are so sure global food needs justify their research that there is no point in further ethical inquiry.^10^

Some combination of these influences may explain why agricultural research institutes have been slow to develop capacity for bioethics. In addition, ethicists in philosophy departments, law schools, and medical bioethics programs may have an oversimplified picture of the landscape for food ethics. The word “agriculture” often connotes the activity of farmers, ranchers, and herders (all characterized broadly as growers). “Food system” more naturally encompasses the seed, breeding, and input firms that supply growers, as well as processing, distribution, and retailing activities occurring beyond the farm gate. Aquaculture is another food production activity that might not come to mind in connection with agriculture. Non-specialists may associate agriculture solely with food production, but many farms and ranches derive income from fiber and other industrial inputs, including materials crucial to the pharmaceutical industry. In addition to fiber production (e.g., cotton, flax, wool, and leather), biologics, biofuel and, in less industrialized settings, traction power are important products that might not immediately be recognized as food-system outputs. Although the biopreservation technologies discussed later generally do relate to food, agricultural research institutes engage in activity reflecting all these endpoints. Here, the terms “agriculture” and “food system” will be used interchangeably.

One additional factor complicates ethical studies of biopreservation in agriculture. Food systems are undergoing the combined effects of technological innovation in many areas. Readers will have some familiarity with recent developments in gene transfer and gene editing that have given rise to both regulatory uncertainty and international controversy.^11^ Less widely appreciated, automated or “driverless” control of farm equipment has been pursued since the 1970s.^12^ GPS-guided tractors and harvesters enable more precise application of agricultural chemicals.^13^ Further innovation in automation and robotics includes the use of AI-enabled sensing technology that can recognize weeds, the use of drones for monitoring the production process, and automated data collection for plant or animal health, feed, or nutrient intake.^14^ Science writer Amanda Little reviews the multiple domains in which innovations are being undertaken, including stem cell and other forms of biotechnology to produce meat, milk, and eggs without the need for raising a living animal. Many of these specific tools are enabled by novel applications of information technology and data analysis.^15^ An unprecedented investment in agricultural innovation by private venture capital since 2010 propels technical change in food systems.^16^

The onslaught of novel tools and techniques under development suggests the potential for large-scale change in food systems over the next decade. At the same time, it will be difficult to isolate the social, economic, or technological factors contributing to the combined effect of these technical developments. It would be foolish to assign causal responsibility for revolutionary change to any one innovation, or even to a single domain of innovation. Change will likely involve multiple applications in several different aspects of food and fiber production, processing, or distribution. Correlatively, assigning ethical responsibility for either beneficial or deleterious outcomes to any particular suite of tools and techniques, such as biopreservation, looks to be a fraught exercise. The cryopreservation innovations discussed below will be but one strand in a complex web of technical changes. A thorough examination of this complexity lies beyond the scope of the present analysis.

## II. Biopreservation in Agriculture and Food

What is biopreservation in the context of agriculture and food? It is useful to begin by noting that techniques for cooling and cold storage of both food organisms and their reproductive materials (seeds, ova, semen, and embryos) have been components of industrialized food systems since the last several decades of the 19th century. William Cronon’s environmental history of the American Midwest singles out the development of refrigerated rail cars as transformative for supply chains that linked stockyards to population centers on the Eastern Seaboard. Cold storage and transport of meats processed in Chicago and Cincinnati reduced the consumer cost for meat consumption, as well as the expansion of retail outlets in New York, Boston, and Philadelphia. The cold chain created new economic opportunities for both animal and feed production throughout the Midwest, leading to the ecological transformation of the Great Plains into a farming region.^17^ Advancement of techniques for biopreservation through cold should be seen as a refinement of tools that have already had prodigious effects on diet, on farm production, and on the wider environment.

Tools and techniques for advanced cryopreservation have three foreseeable points of impact on agriculture and food systems. First, like refrigerated rail cars, elements of the platform for cryopreservation will probably be adapted for post-harvest cold storage and transport. Second, cryopreservation of gametes and embryos will be useful in assisted reproduction of food animals. Relatedly, improved tools for preserving seed, gametes, and embryos should enhance the effectiveness and efficiency of repositories for reproductive material maintained for agricultural purposes. Each of these applications is reviewed in turn.

### A. Post-Harvest Storage and Transport

Advances in isochoric freezing will extend cold chains originally developed through refrigeration. Cooling and chilling of meats, fruits, and vegetables slows the growth of microorganisms, as well as natural ripening processes. This retards spoilage, and as such reduces waste of potentially consumable food products. Cold storage of foods contributes to food safety by inhibiting the growth of toxic microorganisms. However, existing cooling and freezing methods have limitations. All foods contain water, and water expands as it turns into ice under freezing conditions. Conventional forms of cryogenic biopreservation can damage the texture and integrity of plant and animal tissue or byproducts used for food. While some food products withstand temperature change and accompanying expansion and contraction of tissues, others do not. New technologies that enable manipulation of the freezing process during food storage are thus of broad and growing interest.^18^

Isochoric cooling presents one such technology, and uses an ensemble of techniques in place of conventional refrigeration to cool foods below the freezing point (i.e., 0° Celsius). In the most commonplace isochoric cooling process, dubbed “isochoric freezing,” limited ice is allowed to form at the peripheries of the system (apart from the food products), and the expansion of this ice pressurizes the rest of the system to the point that additional growth of ice becomes thermodynamically impossible. In addition to possible medical applications, isochoric techniques are being studied for their potential use on foods that do not respond well to conventional freezing techniques, or whose cold-stored shelf lives are limited by microbial growth. While enhancing the sensory and nutritional quality of stored foods by preventing damaging ice formation at sub-0° C temperatures, this increased pressure (even in mild doses, 5–30 MPa) has also been to shown to reduce or eliminate food-borne pathogens, mold and yeast, and other microbiota, increasing food safety.^19^

The rationale for isochoric methods does not differ from that of conventional freezing, and in some cases isochoric freezing will simply become an alternative to existing techniques. In these applications, cost and possible food safety gains become the primary rationale for isochoric methods. In addition, isochoric freezing may realize energy savings over conventional methods, reducing costs and contributing to the goal of lowering the contribution of food systems to atmospheric change.^20^ However, the innovation also extends the potential for low-temperature biopreservation to foods, such as tomatoes, not otherwise suitable for subzero cold storage without alteration to the taste and texture.^21^ For such foods, isochoric cooling promises a unique approach to maintaining the fresh-like quality that is attractive to many consumers.

### B. Assisted Reproduction

Here, assisted reproduction refers to a cluster of techniques used in farm animal breeding. Though unverified instances may have occurred earlier, the first documented case of *artificial insemination* on dogs occurred in 1784. Sustained research on artificial insemination in food and farm animals accelerated through the early decades of the 20th century. Techniques for inserting sperm into the vagina underwent rapid development beginning in the 1940s. Successful freezing of sperm through the addition of glycerol was perfected in the 1970s. These methods are widely used for breeding in cattle, swine, and poultry today.^22^ *Embryo transfer* involves implantation of fertilized oocytes, often involving *in vitro* fertilization. In food animals, *in vitro* fertilization and embryo transfer accelerate the pace of genetic improvement, rather than addressing reproductive failure in an individual animal. Thus, although the techniques are similar to those used in human reproductive medicine, the purposes differ. Although embryo transfer has an established use in human medicine, Rego describes it as a technology of the future for farmed animals.^23^ Sjunnesson reports that its use in cattle is expanding rapidly, while swine and poultry species continue to face technical challenges that have retarded widespread uptake of embryo transfer to those species.^24^

Reproductive cells and micro-physiological systems can be better transported both for use in research and in the food industry when held at temperatures below freezing. As such, advances in cryogenic biopreservation have the potential to reduce the cost of assisted reproduction in all livestock species.^25^ In addition, the technology could facilitate break-throughs for some areas of commercial food animal production. Although assisted reproduction is common in poultry species, the efficiency of cold storage and transport for semen lags far behind that of the dairy and beef industries.^26^ Reproduction through vaginal insertion of sperm only becomes commercially feasible in food animals when accompanied by cost-effective tests for evaluating semen quality and detecting the estrous cycle, as well as record-keeping for monitoring the performance of individual semen donors and bred females.^27^ Torres and Tiersch argue that the research community is far from being able to solve these problems for fish species. The lack of standards for reproducing and evaluating research on each of these questions is a barrier to progress toward assisted reproduction in aquaculture.^28^ Advanced tools for cryopreservation could simplify these problems, both easing challenges in storage and evaluation of gametes and potentially allowing the industry to move directly to embryo transfer.

### C. Repositories

Nikolai Vavilov (1887–1943) created one of the world’s largest seed collections in St. Petersburg prior to World War II.^29^ The Empire Potato Collection (now the Commonwealth Potato Collection) near Dundee, Scotland was initiated in the 1930s. Both collections were for developing improved crop varieties. The US National Seed Storage laboratory was created in 1958, originally envisioned as depot for conserving the genetic resources of infrequently grown varieties of agricultural crops. The purpose and procedures for seed repositories underwent debate and change over the succeeding decades.^30^ From the perspective of agriculture, the key function is to catalog and preserve genetic constructs that might become useful for crop breeding at some future date. The Commonwealth Potato Collection, for example, was envisioned as a resource that would help agricultural scientists avoid a great famine, such as the one that struck Ireland in the 1840s.^31^

Cold storage has long been used in seed repositories, a prominent feature of Norway’s Svalbard seed bank located within the Arctic Circle. However, frozen seed eventually decays and some species cannot be stored using existing technology. Specimens must be constantly regrown even in the best of conditions. In addition, controversy abounds over access to genetic resources in repositories. Advanced cryopreservation tools thus have the potential to ameliorate, if not totally solve all of these problems by preserving fertility for longer periods, resolving problems associated with freezing and thawing of biological material, and reducing costs for transportation. In addition, the potential for more effective and cost-efficient cryopreservation of gametes and embryos suggests that similar repositories may become feasible for storing the genetic material of animal species.^32^

## III. The Ethical Rationale for Biopreservation in Agriculture and Food

In large measure, agricultural ethics is concerned with what philosophers have called defeasible or *prima facie* goods. Much like medicine and biomedical science, the pace of change in agriculture has accelerated steadily over the last two centuries. There are morally significant reasons to support these changes, but other considerations may countermand these reasons in certain circumstances. As such, agricultural ethics generally begins by categorizing the likely impact of technological innovation in terms of pro and con considerations. Consistent with principlist approaches in bioethics, this inventory is subjected to more thorough evaluation and exchange of arguments.^33^ In this section, we discuss rationales for pursuing advanced cryopreservation techniques in food systems. The succeeding sections discuss reasons why they might prove to be problematic in specific situations.

As just discussed, innovative applications of biopreservation extend practices in current use, primarily by making techniques for cold storage and transport more broadly applicable. As such, the rationale for further advances in cryopreservation derives from practices in use for over a century. Storage and transport of seed, semen, ova, and zygotes facilitate plant and animal breeding. Breeding, in turn, is a key tool for increasing farm-level productivity, making more efficient use of soil and water resources, and remediating unwanted environmental impact from agriculture. Although productivity-increasing innovations in agriculture have been criticized as being motivated by profit seeking, ethical arguments can be marshalled in their defense. In the context of agriculture, preservation of a diverse pool of alleles also provides a source for future breeding to address challenges from disease or change in abiotic elements (including climate change) in agricultural ecosystems.

In fact, rationales for advances in cryopreservation overlap with the rationale for increasing productivity in agriculture more generally. First, increasing the food supply supports the basic human right to food and increases human welfare through reducing the cost of food and fiber goods.^34^ Furthermore, as a potentially renewable resource, agricultural production promotes sustainable consumption when plant and animal production becomes less resource-consumptive or reduces impact on the broader, non-farm environment.^35^ Third, agriculture is an important source of livelihood, especially in less industrialized regions, where as much as 80% of the population may be employed in agriculture. The livelihood arguments take on ethical significance because the agricultural sector is critical to the economic development of many rural areas, where plant and animal production may support social services for the non-farm population.^36^ Agriculture thus contributes to overall economic health in less industrialized countries, and may be a critical source of foreign exchange.^37^ In summary, agricultural technologies that strengthen livelihoods contribute directly to the farm population’s welfare and indirectly to the entire rural economy.^38^

It is also important to stress the link between agricultural production and distributive justice. On a global level, between 50% and 70% of people in extreme poverty (less than 1 euro per day) are farmers or derive their livelihoods from other food system employment. As such, supporting agriculture has a distributive benefit beyond its overall contribution to welfare.^39^ There are also ethically significant redistributive benefits for consumers. Since the food budget consumes a larger portion of income for the less well off, technology that reduces food cost supports the Rawlsian criterion of disproportionately benefiting the worst-off group.^40^

This summary of positive outcomes functions as a presumptive ethical argument in favor of productivity-increasing innovation in food systems. It captures the rationale for improvement in the biopreservation methods currently in use. It is a standard argument for achieving greater efficiencies in using scarce resources such as land and water and for reducing unwanted effects of agriculture, including pollution, environmental degradation, and biodiversity loss. Résumés like this appear frequently in journals and other publications on current work in the agricultural sciences. These summaries stress future benefits and rarely discuss considerations that might qualify the endorsement of technology development.^41^ Papers that reference the benefits of increasing productivity in food systems rarely represent the argument as ethical in nature. Nevertheless, they can be interpreted as a loose adaptation of Pareto-better consequentialism: benefits that exceed costs are presumed to offset or override other considerations (such as the distribution of benefit and cost), even if these countervailing factors are not explicitly discussed. In a more complete ethical evaluation, reasons to qualify or vitiate projections of future benefits are given explicit consideration.

## IV. Substantive Ethical Concerns for Technological Innovation in Agriculture and Food

In sum, advanced cryopreservation techniques promise to increase productivity by reducing waste and by introducing more cost-efficient means for genetic improvement in food animals. Critics of innovation in agriculture and food systems point to harmful outcomes that have occurred in specifiable instances, countering the suggestion that the benefits of increasing productivity will outweigh harm in every case. Pro-innovation arguments should be situated in a broader ethical framework that also considers objections. Here, it will be useful to consolidate the ethical argumentation that might counter this broadly supportive rationale for increasing productivity into manageable categories. Succinctly, opposition to technological innovations derives from (a) risk of harm to third parties, including non-humans; (b) ecological integrity and environmental justice; and (c) socioeconomic impact on the farming population. This classification reflects the practice of scholarly communities, rather than moral, conceptual, or ontological categories. Unwanted outcomes such as toxicity or malnutrition align well with medically oriented bioethics. Ethical considerations relating to ecological impact and environmental justice are topics in environmental philosophy, broadly conceived. Scholars in rural studies have produced a significant literature on socioeconomic impact in food systems, but the topic receives relatively little attention from normative theorists. Issues such as the role of agriculture in colonization might be located in all three categories. Again, this classification scheme is for the purposes of summarizing a complex literature, rather than establishing robust ethical or epistemological categories.

Non-maleficence provides the ethical rationale for constraining unintended consequences harmful to human health, with significant bioethical literatures on toxicity and malnutrition.^42^ Philosophical controversy also arises in food systems over epistemic issues in the measurement or management of risks. Concepts poorly adapted to crop or animal production. Most obviously, the development of synthetic fertilizer allowed farm production to expand into areas with poor soil.^45^ Critics of genetically engineered maize varieties expressed the fear that they would displace irreplaceable sources of genetic diversity if they were grown in the center of origin for maize.^46^ Environmental injustice occurs when marginalized groups bear a disproportionate share of the burden from land use or administration of policy. The most obvious case in agriculture was the US Department of Agriculture’s multi-decade denial of service to Black such as threshold of exposure, *de minimus* risk, and chemical hormesis (the shift from beneficial to toxic impact at varying levels of exposure) are open to multiple interpretations.^43^ Such epistemological issues can have policy implications. Language requiring the Food and Drug Administration (FDA) to apply an especially rigorous standard to possible carcinogens led to a torturous series of decisions on artificial sweeteners.^44^ As discussed later, scientific uncertainty interacts with other sources of ethical concern in mobilizing public outrage over agriculture and food system innovations. Philosophical debates overextending the principle of non-maleficence beyond human beings are also relevant to agriculture. Here, the philosophical questions of non-maleficence bleed seamlessly into questions familiar to the ethics of animal use.

The second category encompasses more broadly defined environmental issues. New agricultural technology can make it possible to farm areas hitherto farmers throughout the South, along with exploitation of Black sharecroppers and contract workers by white landowners.^47^ However, technology can become implicated in environmental justice. In the 1930s, cotton growers may have adopted recently developed mechanical harvesters in order to subvert the Franklin D. Roosevelt Administration’s attempt to reform southern sharecropping.^48^ Mechanization of the California tomato industry in the 1960s displaced the labor of Mexican workers.^49^

This sort of issue in environmental justice overlaps with socioeconomic issues. In addition to displacing a racially marginalized Latino workforce, the tomato harvester precipitated a rapid transformation of the ownership structure in the California canned-tomato industry. The harvester could not be operated in a cost-effective manner on small acreage. Within a few years of its implementation, hundreds of California producers no longer grew tomatoes, converting tomato farming into an industry with a handful of large farms.^50^ This type of structural transformation often occurs in response to innovations that reduce costs in farm production. Even without a scale bias, such as occurred with the tomato harvester, better-off farmers tend to be among the first to utilize cost-saving innovations. They realize windfall profits during this period. The supply curve (and hence the market price) shifts when the technology is widely adopted, so late adopters never realize the windfall benefits of early users. Meanwhile, early adopters use their windfall to purchase land holdings of late adopters driven to bankruptcy. The result is consolidation: an economic structure that leads to fewer and larger farms with each cycle of innovation. Willard Cochrane, the farm economist who described this phenomenon, called it the technology treadmill, arguing that it would be a pervasive problem for productivity-increasing innovations.^51^ Even more generally, the global spread of European-style household farming systems displaced complex indigenous food systems.^52^ Some view agribusiness sponsorship of technical innovation as a continuation of colonial oppression.^53^

Socioeconomic impacts also become important because there is a history assigning special moral significance to the farming population. On one hand, agriculture and other elements of the food system make significant contributions to the domestic economy in most nations. Protecting the economic interests of the farm sector has been politically popular throughout much of human history. On the other hand, these pecuniary interests derive support from complex (and sometimes dubious) claims about the moral character of rural people, their patriotism, their environmental stewardship, and their willingness to shoulder the burdens of citizenship. Farm families are said to be an especially significant source of national or cultural identity. Ironically, views linking farming to exceptionalist visions of national character are quite widespread among global cultures.^54^ Although some analysts view agrarianist philosophies as a peculiar source of American exceptionalism,^55^ similar arguments are made almost everywhere. Widespread economic failure of farmers and decline in the number of farms is thus regarded as morally problematic. A similar change in the number of people practicing other professions would be unlikely to provoke moral outrage.

In conclusion, this section has offered an illustrative, but not exhaustive, account of competing goods having the potential to counter the pro-innovation argument. To repeat, that argument stressed expansion of the global food supply, mitigation of harmful environmental impact, and cost savings for all actors in food savings, but especially for impoverished consumers who expend a greater proportion of their income on food. The potential harm to third parties, negative environmental impacts, or socioeconomic disruption stand as reasons why these beneficial outcomes might overridden in certain cases. Completing the pro-innovation argument thus requires a response explaining how benefits outweigh or offset potential harms, or how aspects of the technology or its manner of introduction will accommodate the concerns noted in the critiques. Looking ahead to procedural and institutional issues, the failure to make any clear ethical response to concerns then becomes both a source of distrust, and an ethical issue in its own right.

## V. Ethical Issues for Biopreservation in Aquaculture

Where do applications of advanced cryopreservation sit within this landscape? Here we consider just one case: advanced cryopreservation for improving aquaculture. Increasing productivity in aquaculture would lower costs to consumers, plausibly serving the ethical goals of reducing hunger and increasing welfare, especially among less wealthy consumers. Increasing the use of fish species in human diets promises to lower the environmental footprint of food production owing to superior feed-to-food conversion when compared to other food species. In comparison to fishing wild species, aquaculture is expected to have lower impact on biodiversity and ecosystem services.^56^ Arguments for increasing aquaculture productivity are especially salient given the uncertainties of future food production resulting from climate change. The Food and Agriculture Organization (FAO) and the Intergovernmental Panel on Climate Change (IPCC) are both predicting global shortages of food by 2050 owing to the loss of arable land from rising seawaters and destabilization of rainfall patterns.^57^ Thus, improving the cost-effectiveness of biopreservation in aquaculture is supported by an array of ethical arguments that have been developed in connection with other innovations in food production.

In a 2000 overview of ethical issues for cryopreservation in aquaculture, Wachtel and Tiersch review ethical debates on storage of human gametes and embryos, and then list 15 issues where biopreservation creates ethical quandaries for aquaculture. Many arise from the way enhancing storage makes the outcome of assisted reproduction less certain. Social conventions for use may change, making assessment of the impact on industry structure or small-scale producers unclear. The ecological context itself may change, making the environmental fate of stored specimens difficult to predict. In this connection, organisms produced from biopreserved materials could become invasive or reintroduce diseases into future populations. Wachtel and Tiersch suggest that cryopreservation may be more appropriate for conservation than for food production, in part because profit-seeking may lead to unwanted change in the socioeconomic structure of the food sector. They also note that an infrastructure for monitoring the impact of cryopreservation needs to be created.^58^

The issues reviewed by Wachtel and Tiersch speak to conservation as well as food production. For example, the chance that a biopreserved aquatic species might become invasive in the future is most pertinent to environmental release of samples stored over a span of many decades. In a food production context, stored samples would be used in breeding, where the genetics of a biopreserved specimen will be integrated with those of breeds in current use. Nevertheless, Wachtel and Tiersch’s list of concerns falls into the category of unknown and potentially uncontrollable environmental impacts from food production. In this connection, it is important to note that ocean-pen forms of agriculture have already been the focus of critique by environmental ethicists.^59^ In addition, the genetically engineered AquaBounty salmon became one of the most aggressively contested GMOs.^60^

Turning to the ethics of socioeconomic impact, the global structure of aquaculture becomes a prominent focus. Current ethical debates on aquaculture compare the environmental and animal welfare impact of ocean pens vs. production in tanks, which may be ponds of several acres in surface area. Tank-based production is used by smallholders to generate a viable income from a land base too small to support the household from traditional farming, especially in Asia.^61^ Aquaponic systems operate at an even smaller scale. They are being evaluated for use as food or income supplements in developing as well as industrial economies.^62^ At this point, further discussion of the socioeconomic ethics of advanced cryopreservation in aquaculture would be speculative. However, the existence of these smallholder systems suggests that in addition to the burdens just noted, the ethics of advanced cryopreservation should also include an evaluation of the potential impact on the profitability of smaller scale production, smallholder access to the technology, and the potential for scale bias, as seen in the case of the tomato harvester. The potential for Cochrane’s technology treadmill cannot be excluded preemptively.

Finally, the sheer potential for socioeconomic impacts attains ethical significance from the larger political context in which biopreservation technologies are being developed. A 2017 study led by Joanna Radin and Emma Kowal reviewed the political ecology of cryopreservation. Contributors link the technology to the growth of biopolitics: a centuries-long process in which aspects of life are laid open to ever-greater forms of subjection and political control. Few make direct mention of biopreservation in food and agriculture, and none mentions aquaculture.^63^ However, Rebecca J. H. Woods considers 19th century food refrigeration to be an important contributor to an objectionable growth in social control.^64^ Frédéric Keck links public health initiatives focused on controlling viral disease to the emergence of the industrialized poultry industry.^65^ Other contributors stress biomedical or conservation applications of cryopreservation. This study could be read as laying down the intellectual foundations for a movement of social resistance that would, like the anti-GMO movement studied by Schurman and Munro, unite activists focused on biomedicine, biodiversity, and more abstruse religious and metaphysical concerns. Whether this would spill over into sustained concern with cryopreservation in food systems is impossible to tell.

## VI. Social and Political Resistance to Innovation in Agriculture and Food Systems

Technological innovations in food and agriculture became increasingly controversial during the 20th century. By century’s end, debates over the safety of food additives, the environmental impact of pesticides, the treatment of livestock, the displacement of farm labor, and the decline of small farms were beginning to merge into a general distrust of agribusiness and the scientific disciplines that support it. The signature event in this transition was outcry over so-called GMOs — the acronym used to classify plant varieties and animal breeds developed using the tools of recombinant DNA. The GMO controversy is useful in the present context for two reasons. First, it illustrates how risk issues, environmental impacts, and socioeconomic transformations combine to generate a whole that is larger than the sum of its parts. Second, the controversy serves to bridge these substantive ethical questions to the procedural issues noted earlier in this paper.

Critics raised each health, environmental, and socioeconomic ethical concern against GMOs between 2000 and 2010, the period of greatest controversy. A coalition of social activists, each of whom might have been motivated by just one of these concerns (or by a general opposition to genetic manipulation and the fear it might be used on human beings), coordinated protests and public relations activities highlighting potential ethical concerns. Though these activists may have been least concerned about the ethics of food safety, the climate of uncertainty created by their activity led to a broad public perception that supporters of the technology were unwilling to take ethical concerns seriously. Opponents of gene technology parlayed epistemological questions into a precautionary ethic suggesting that food safety testing and regulation of the technology might be inadequate.^66^ Labeling of GMOs was suggested as an informed consent approach to both food safety and socioeconomic risks, acknowledging the consumer’s right to avoid putative hazards, even in the absence of scientific support.^67^ At a single stroke, the opportunity to move public opinion and score political victories united a disparate group of activists, each of whom had distinct ethical concerns, while the apparent unwillingness to engage seriously with these concerns was translated into a comprehensive worry over the agricultural establishment’s commitment to safety and the public interest.

Resistance to GMOs serves as a cautionary tale for any innovation in food system technology. Gene technologies have aspects that make them especially fecund attractors of ethical opposition, but they are not the only innovations to have sparked ethical critique. As noted already, mechanization was challenged for its impact on labor and land tenure. Biofuel technology has been an especially frequent target of ethical criticism.^68^ In the 2000s, critics of GMOs also leveled attacks on nanotechnologies.^69^ Indeed, Susanne Friedberg’s study of innovations for storage and transport of foods documents a history of controversy in which purveyors of older technologies such as harvesting ice or salting impugned refrigeration by suggesting it created the opportunity to conceal flaws in stored and transported foods. At the same time, Friedberg notes how once new techniques were accepted, the consuming public’s understanding of freshness was itself subject to change.^70^

Ethical objections to irradiation of perishable foods are of most direct relevance to advanced cryopreservation. Ionizing radiation destroys microorganisms present in all unprocessed foods, extending the shelf life without refrigeration. Although scientific evidence for the safety of irradiation is overwhelming, the technology has been opposed as anti-farmer in its expected impact on the price of fresh produce and as the food industry’s attempt to gain advantage over farmers. These social impacts are conjoined with public fears over radiation risks, in general.^71^ A precautionary ethic counters the scientific defense, noting multiple sources of uncertainty in risk assessments. The scientific defense of irradiation has also been insufficiently attentive to reasonable consumer concerns. As such, there is a procedural argument directed less toward the techniques of food irradiation than to the failure to solicit and accommodate public input in the innovation process.^72^ Mandatory labeling is required for irradiated foods in the United States.^73^

This history of social and political controversy has ethical implications. Focusing again on the case of aquaculture, the existence of prior debate over innovations ranging from net pens to AquaBounty salmon sets the stage for additional innovations to become embroiled in ethical controversy.^74^ Thus, developing more advanced cryopreservation for aquaculture must include strategies for (1) evaluating and potentially adjusting the technology to account for potential social and environmental impacts, and (2) anticipating resistance to the technology and engaging with persons or groups that voice objections. It is important to see that that addressing (1) does not automatically satisfy ethical responsibilities to engage with critics. Papers on environmental or social impact buried in the scientific literature make an inadequate response. There is an additional responsibility to develop an explicit ethical argument showing that expected benefit from increased productivity offsets or outweighs other ethical concerns, and to bring this argument to an audience beyond the scientific community. This is not to say that critics must be appeased or convinced. Yet failing to attempt a reasonably complete public airing of the rationale for innovation that explicitly addresses the argument of opponents cannot be said to satisfy the ethical requirements for legitimate technical change.

Controversies over mechanization, irradiation, and genetic engineering in agriculture suggest ethical issues lying beyond substantive ethical issues such as guaranteeing the right to food, the challenges of climate change, and feeding future generations. These substantive concerns may be offset by competing goods: avoiding harm to third parties, the complexity of environmental impact, and the history of socio-economic displacement. Yet, there are also procedural questions about how substantive issues are addressed within the agricultural sciences. The ethical evaluation of technical innovation requires interdisciplinary research tailored to specific tools and techniques and to the specific sector of food and agriculture in which the tools will be applied. Normative, philosophical expertise contributes conceptual analysis, but it needs to be conversant with the basic issues raised by innovation in the food and agricultural sector. Arguably, multidisciplinary bioethics programs in medical schools arose to address comparable problems in biomedicine. A capacity for analyzing and debating substantive issues was created. While many substantive ethical issues in medicine are far from being resolved, bioethics programs (as well as forums such as the various Presidential bioethics commissions) function to satisfy the need for a procedure that addresses issues in a systematic and responsible fashion. Nothing remotely comparable exists within US agricultural science organizations. In short, the failure to develop a capacity for addressing substantive ethical issues in agriculture and food systems is itself an ethical issue plaguing the innovation process.

## Conclusion

The ethical pros and cons of using advanced cryopreservation in the food system are not unprecedented. Improved tools for biopreservation join many agricultural and food technologies in promising to assure a food supply adequate for the global population, while reducing energy use and environmental impact, including the release of climate-forcing gasses. At the same time, the history of mechanical, chemical, and biotechnological innovations is one of controversy and contestation that might have been more effectively and fairly adjudicated by an anticipatory ethical review. The future benefits of isochoric freezing, enhancements in assisted reproduction, and improved storage of genetic material are more likely to be realized if they are pursued in conjunction with a bioethical review. Such a review would take account of the potential for ethical pitfalls, explore opportunities for avoiding or mitigating unwanted outcomes, and articulate the rationale for navigating the landscape of competing goods in one fashion, rather than another.

However, with this observation we return to a point noted at the outset. The institutional capacity for any ethical review of technological innovations in food and agriculture is limited. On one hand, the capability to undertake ethical analysis is constrained by the dearth of resources allocated by colleges of agriculture and through key funding organizations, such as the US Department of Agriculture or the Gates Foundation. On the other hand, bioethicists, environmental philosophers, and other scholars of ethics have themselves given little attention to the organization and operation of food systems, or to the ethical issues and controversies arising therein. As such, the overriding issue is not one unique to innovations in biopreservation. It is the moral imperative to give this complex, but essential, element of industrial society and contemporary life the attention it so richly deserves.
